# Application of Transcriptomics to Compare the Carbohydrate Active Enzymes That Are Expressed by Diverse Genera of Anaerobic Fungi to Degrade Plant Cell Wall Carbohydrates

**DOI:** 10.3389/fmicb.2018.01581

**Published:** 2018-07-16

**Authors:** Robert J. Gruninger, Thi T. M. Nguyen, Ian D. Reid, Jay L. Yanke, Pan Wang, Denis W. Abbott, Adrian Tsang, Tim McAllister

**Affiliations:** ^1^Lethbridge Research and Development Centre, Agriculture and Agri-Foods Canada, Lethbridge, AB, Canada; ^2^Centre for Structural and Functional Genomics, Concordia University, Montreal, QC, Canada

**Keywords:** rumen, neocallimastigomycota, CAZome, carbohydrate active enzymes (CAZymes), anaerobic fungi

## Abstract

The efficiency with which the anaerobic fungi (phylum Neocallimastigomycota) degrade plant biomass is well-recognized and in recent years has received renewed interest. To further understand the biological mechanisms that are utilized by the rumen anaerobic fungi to break down lignocellulose, we have used a transcriptomic approach to examine carbohydrate digestion by *Neocallimastix frontalis, Piromyces rhizinflata, Orpinomyces joyonii, and Anaeromyces mucronatus* cultured on several carbon sources. The number of predicted unique transcripts ranged from 6,633 to 12,751. Pfam domains were identified in 62–70% of the fungal proteins and were linked to gene ontology terms to infer the biological function of the transcripts. Most of the predicted functions are consistent across species suggesting a similar overall strategy evolved for successful colonization of the rumen. However, the presence of differential profiles in enzyme classes suggests that there may be also be niche specialization. All fungal species were found to express an extensive array of transcripts encoding carbohydrate active enzymes (CAZymes) ranging from 8.3 to 11.3% of the transcriptome. CAZyme families involved in hemicellulose digestion were the most abundant across all four fungi. This study provides additional insight into how anaerobic fungi have evolved to become specialists at breaking down the plant cell wall in the complex and, strictly anaerobic rumen ecosystem.

## Introduction

Ruminant and hindgut fermenting herbivores harbor a diverse microbial community that break down ingested plant with potent cellulolytic and hemicellulolytic enzymes. The anaerobic fungi (Phylum Neocallimastigomycota) are known to be essential members of this community and play a key role in the colonization and degradation of the plant cell wall (Gruninger et al., 2014; Edwards et al., 2017). The importance of the anaerobic fungi to host metabolism was demonstrated by a decrease in voluntary feed intake and dry matter degradation following selective removal of Neocallimastigomycota from the rumen of sheep (Gordon and Phillips, 1993). These observations were taken as evidence that digestion of recalcitrant fiber in the rumen was impaired in animals that lacked anaerobic fungi (Gordon and Phillips, 1993). Anaerobic fungi degrade lignocellulose using a large portfolio of Carbohydrate-Active enZymes (CAZymes) and penetrating hyphae that physically disrupt the ultrastructure of the plant cell wall (Youssef et al., 2013; Solomon et al., 2016; Haitjema et al., 2017). This action is hypothesized to increase the surface area for bacterial colonization and further enzymatic digestion. In anaerobic fungi, CAZymes can be found as both free enzymes and in a multiprotein complex called the cellulosome (Haitjema et al., 2017). The genome sequencing of four species of Neocallimastigomycota suggests that many of these CAZymes have been acquired by horizontal gene transfer from rumen bacteria (Youssef et al., 2013; Haitjema et al., 2017). The extensive CAZyme repertoire, cellulosome, and extracellular proteases produced by Neocallimastigomycetes may help these microbes compete with other rumen inhabitants for limited nutrients (Youssef et al., 2013; Haitjema et al., 2017).

Anaerobic fungi diverged early from the lineage that led to the Ascomycota and Basidiomycota, and are most closely related to the chytrids (Phylum Chydridomycota) (Hibbett et al., 2007). The fungi within the phylum Neocallimastigomycota display both monocentric and polycentric morphologies and were originally classified into six well-established genera. The monocentric genera are *Neocallimastix, Piromyces*, and *Caecomyces*, and the polycentric genera are *Orpinomyces, Anaeromyces*, and *Cyllamyces*. Within the last 3 years, three additional genera have been described: *Oontomyces* (Dagar et al., 2015), *Buwchfawromyces* (Callaghan et al., 2015), and *Pecoramyces* (Hanafy et al., 2017). *Pecoramyces* is closely related to *Orpinomyces* and was only recently reclassified from its original designation, *Orpinomyces* sp. C1A (Youssef et al., 2013). Metagenomic studies have revealed many more phylogenetically distinct clades that are not represented by cultured isolates (Liggenstoffer et al., 2010; Koetschan et al., 2014).

Until recently, the fastidious nature of the anaerobic fungi, high AT content, and the highly repetitive nature of their DNA have hampered genomic research in these microbes. The high AT content and long stretches of repetitive sequence has made assembling their genomes difficult. Recently a comparative genomics study examined the assembled genomes of 3 genera within Neocallimastigomycota (Haitjema et al., 2017). This study along with the analysis of the *Orpinomyces* C1A genome has greatly enhanced the understanding of the evolution of these fungi and the adaptations they have acquired to survive in a competitive anaerobic environment. Some of these adapatations include the replacement of ergesterol with tetrahymanol in the plasma membrane, the dependence on mixed acid fermentation for pyruvate metabolism (Youssef et al., 2013), and the use of hydrogenosomes instead of mitochondria for ATP generation (Yarlett et al., 1986). The existence of cellulosomes in Neocallimastigomycota has long been proposed, but the identity of the scaffoldin remained elusive. The recent work of Haitjema et al. (2017) used a combination of genomics and proteomics to identify the scaffoldin in Neocallimastigomycota and characterize the the dockerin-scaffoldin interaction (Haitjema et al., 2017).

Transcriptomic studies provide a functional view of an organism by identifying the genes actively expressed under a set of metabolic conditions. Transcriptomics with anaerobic fungi cultures avoids assembling intron sequences and the longer repetitive reads, which greatly complicate genome assembly. To date, five transcriptome studies of AF have been published (Wang et al., 2011; Youssef et al., 2013; Couger et al., 2015; Solomon et al., 2016; Henske et al., 2017). To further understand the biological mechanisms utilized by the anaerobic fungi to break down lignocellulose, we have conducted a transcriptomic study of carbohydrate digestion by *Neocallimastix frontalis, Piromyces rhizinflata, Orpinomyces joyonii*, and *Anaeromyces mucronatus* when cultured on structurally distinct plant cell wall substrates. These data were used to undertake a detailed analysis of the entire contingent of CAZymes expressed by these fungi (i.e., their CAZomes). By comparing the profiles of the anaerobic fungi, aerobic fungi, and rumen and non-rumen bacteria, we also provide insight into the differential mechanisms employed by Neocallimastigomycetes to degrade lignocellulose.

## Methods

### Fungal isolates and culturing

All strains were started from stock cultures stored in liquid nitrogen at the Agriculture and Agri-Food Canada Research Centre in Lethbridge Alberta Canada. *Anaeromyces mucronatus* YE505 was originally isolated from an elk, *Orpinomyces joyonii* SG4 was originally isolated from sheep, *Neocallimastix frontalis* 27 was originally isolated from cattle and *Piromyces rhizinflata* YM600 was originally isolated from moose (Hausner et al., 2000). Fungal biomass was produced under strict anaerobic conditions using the Hungate method for preparing anaerobic media and growth of anaerobes (Wolfe, 2011). The headspace above the media consisted of anaerobic CO_2_ that had been scrubbed of oxygen by passing over a reduced copper column heated to 370°C. Fungi were grown without agitation in at 39°C in 100 mL of Lowe's semi-defined medium (Table S1; Lowe et al., 1985) supplemented with 1 g of one of the following carbon sources: (1) glucose (Sigma-Aldrich, Oakville ON), (2) filter paper, (3) oat spelt xylan (Sigma-Aldrich, Oakville ON), (4) barley straw, (5) rice straw or (6) alfalfa hay. RNA from three biological replicates of each fungal species was sequenced for all carbon sources. Circles of filter paper obtained using a hole-punch were used for conditions containing this carbon source. Straw and alfalfa was ground to 4 mm with a Wiley-mill (Thomas Scientific, Swedesboro NJ) and passed over a screen to remove fines. Only *A. mucronatus* and *P. rhizinflata* were grown on rice straw and only *O. joyonii* and *N. frontalis* were grown on filter paper.

### RNA extraction and sequencing

After 72 h of growth, the fungal mycelia were separated from growth media and harvested using a vacuum filter fitted with a Büchner funnel and Whatmann quantitative 50 fast flow filter paper (GE Lifesciences, Mississauga ON). Fungal mycelia were then carefully separated from growth substrate that had not been colonize and frozen in liquid nitrogen. The frozen mycelia were ground to a fine powder using a mortar and pestle and re-suspended in 1 mL of Trizol reagent per 100 mg of fungi according to the specifications of the manufacturer (Life Technologies Inc. Burlington, ON). Total RNA was isolated from Trizol according to the manufacturer's instructions. After isolation from trizol, the total RNA was further purified using a column based purification with the MEGAclear kit from Ambion (Life Technologies Inc. Burlington, ON). RNA quality was monitored using an Agilent Bioanalyzer 2100 by running samples on a RNA 6000 nano chip. All samples that were sequenced had an RNA integrity number >7.5. Messenger RNA was isolated from the total RNA through a poly-A enrichment step prior to generating the sequencing libraries. Strand-specific RNA libraries were prepared with the Illumina TruSeq Stranded mRNA kit (Illumina, San Diego CA) at the McGill University / Genome Quebec Innovation Centre. Sequencing was done using an Illumina HiSeq 2000 instrument at the McGill University/Genome Quebec Innovation Centre. The resulting single-end 100-nt reads have been deposited in the NCBI Short Reads Archive with SRA accession number SRP135260.

### Read cleaning and assembly

The RNA-Seq reads were trimmed of adapters and low-quality sequence with Trimmomatic (Bolger et al., 2014) or Skewer (Jiang et al., 2014). Reads derived from ribosomal RNA were removed with SortMeRNA (Kopylova et al., 2012). All the reads in the triplicate samples from the same species were pooled and then subsampled by digital normalization with a 7x coverage. The cleaned and normalized reads were assembled with Megahit (Li et al., 2015) using default parameters except—k-skip 6. Contigs were oriented by mapping the reads to them with Bowtie2 (Langmead and Salzberg, 2012), counting the reads that mapped to each strand, and reverse-complementing the contig sequences when necessary. The reads were then mapped to the oriented contigs in strand-specific mode with Bowtie2 and trimmed/split at coverage gaps. Read coverage was estimated by aligning the reads to the contig sequences with Bowtie2 and Salmon (Patro et al., 2017). Errors in the nucleotide sequences of the contigs were revealed by mapping the reads to the contigs with Bowtie 2. Significant differences between the contig sequence and the consensus of the aligned read sequences were identified and corrected with freebayes (Garrison and Marth, 2012). This correction process was iterated until the number of proposed corrections per transcriptome was < 100. The contigs were sorted in increasing order of mapped read count, and their cumulative read count was calculated. Contigs in the low-coverage end of the list with cumulative read counts < 0.5% of the total read count were discarded.

### Contig clustering and filtering

To reduce redundancy, the contigs were clustered with usearch (Edgar, 2010) at 97% minimum nucleotide identity over 80% of the shorter sequence and replaced by the cluster consensus sequences. The alignments of the clustered proteins were transferred to the contigs that encoded them, and a consensus sequence was calculated for each group of contigs corresponding to a protein cluster. These consensus contigs were trimmed at gaps in read coverage as above.

To identify alternatively-spliced transcript isoforms that had been merged during assembly or clustering, a spliced alignment of the reads to the open reading frames (ORF) cluster contigs was performed with two passes of STAR (Dobin et al., 2013). Splice junctions with known donor-acceptor sequences (GT-AT, GC-AG, AT-AC) that occurred in at least two non-identical reads were used to generate potential alternative splicing isoforms from the ORF cluster contigs. Potential isoforms that lacked an ORF at least 150 bases long were discarded. The expression of the ORF cluster contigs and their potential isoforms was estimated with Salmon (Patro et al., 2017). Potential isoforms that were expressed at 1 Transcript Per Million or higher, or that contributed more than 10% of the total expression of all isoforms of their parent contig, were added to the transcriptomes.

### Cross mapping of RNA-seq reads to predicted transcripts

The predicted transcripts of *A. robustus* S4 (Haitjema et al., 2017; Mondo et al., 2017) and *P. ruminatium* C1A (Youssef et al., 2013; Couger et al., 2015) were downloaded from the Joint Genome Institute website (genome.jgi.doe.gov). The number and location of RNA-Seq reads from *A. mucronatus* and *O. joyonii* that mapped to these transcripts were determined with Salmon version 0.9.1 (Patro et al., 2017). For comparison, RNA-Seq reads from *A. robustus* dowloaded from the Sequence Read Archive (www.ncbi.nlm.nih.gov/sra), runs SRR4063398 and SRR4063399, were mapped to the *A. robustus* transcripts in the same way. The mapped read counts were summed over the transcripts in each orthogroup, divided by the number of transcripts and multiplied by 10^9^. This was then divided by the number of RNA-Seq reads to give mapped reads per transcript per billion reads for each orthogroup. The results were visualized with the R library ggplot2 (cran.r-project.org/web/packages/ggplot2/index.html).

### Open reading frame and protein prediction

To detect ORFs the contig nucleotide sequences were translated into protein sequences in all three forward frames and broken into ORFs at stop codons. ORFs shorter than 210 nucleotides were discarded and the set of non-overlapping ORFs with greatest total length from each contig was selected. ORFs that ended with a stop codon and included a start codon more than 210 nucleotides upstream of the stop were considered to be full-length. Final ORF and protein sequences were predicted from the polished contig sequences and this was used for functional annotation.

### Transcriptome quality and completeness

The qualities of the transcriptome assemblies were evaluated with TransRate (Smith-Unna et al., 2016). Their completeness was estimated with the BUSCO program version 1.b1 (Simão et al., 2015) using a modified version of the supplied fungal protein family set. Because the Neocallimastigomycota lack mitochondria, 155 protein families that were annotated by UniprotKB (Uniprot, 2012) as functioning in mitochondria were removed from the set. Protein families that BUSCO reported to be duplicated in more than one transcriptome were examined using hmmalign and phylogenetic analysis. These proteins and the family consensus sequences were aligned using hmmalign version 3.1b2 (http://hmmer.org/) with the trim option to exclude subsequences not covered by the HMM. Approximately maximum likelihood phylogenetic trees were calculated from the multiple sequence alignments with FastTree (Price et al., 2010). The trees were parsed with a custom Python script to identify clades containing proteins from two or more species and pairs of protein sequences from the same species with higher similarity to each other than to proteins from other species.

### Functional annotation of proteins

Functions of the predicted proteins were inferred from their best hits in blastp (Altschul et al., 1990) searches against the non-redundant (nr) protein database from NCBI (http://www.ncbi.nlm.nih.gov/) and SwissProt database at a cut-off e-value of 1 × 10^−20^. Domain information and gene ontology (GO) terms (Ashburner et al., 2000; Gene Ontology, 2015) were obtained by running InterProScan version 5.26.65.0 (Jones et al., 2014). The predicted proteins were also compared to fungal protein sequences in the OrthoDB8 database (Kriventseva et al., 2015) with blastp at an e-value cutoff of 1 × 10^−50^. The Eukaryotic Ortholog Group (EOG) and the EOG annotation for the best three hits of each predicted protein were recorded.Functional annotation for all transcripts can be found in Supplementary Datasheets 1–4.

### Identification and analysis of CAZymes

Carbohydrate-Active Enzyme (CAZyme) families (Lombard et al., 2014) were detected by running hmmscan from the HMMER v3.1b1 package (http://hmmer.org/) with an e-value cutoff of 1 × 10^−5^. Most of the Hidden Markov models (HMMs) were acquired from the dbCAN v6.0 database (Yin et al., 2012). For carbohydrate-binding module family 10 (CBM10), the HMM was downloaded from the PFAM database (Finn et al., 2016). For carbohydrate esterase 1 (CE1) and glycosyl hydrolase 74 family (GH74), in-house HMMs were constructed using experimentally characterized CE1 and GH74 proteins in the CAZy database. Among overlapping modules, the one with the lowest e-value was retained. Domains covering <30% of the HMM were discarded.

### Analysis of CAZy family distribution in different groups of organisms

The distribution of CAZy families amongst anaerobic fungi, aerobic fungi, rumen bacteria, and non-rumen bacteria were examined. The anaerobic fungal group consisted of the four anaerobic fungal species from this study and the publicly available transcriptomes from *Pecoramyces* sp.C1A (Couger et al., 2015), *Anaeromyces robustus* and *Piromyces* sp. “finn” (Solomon et al., 2016). Protein sequences of the aerobic fungi were downloaded from MycoCosm, the fungal genomics resource at DOE's Joint Genome Institute (JGI) (https://img.jgi.doe.gov/). The bacterial protein sequence data sets were obtained from NCBI (https://www.ncbi.nlm.nih.gov/) and the Integrated Microbial Genomes (IMG) system at JGI. The analysis included 104 aerobic fungi, 49 rumen bacteria and 53 non-rumen bacteria. These microbes were selected based on their ability to metabolize the plant cell wall carbohydrates cellulose, hemicellulose, pectin and xylan. The microbes were selected to provide broad view of plant cell wall digestion by evolutionarily diverse organisms inhabiting different environments. A complete listing of the organisms included in this analysis can be found in Table S2. CAZyme modules from these organisms were identified using the method described above to examine the anaerobic fungal transcriptomes. A total of 139 GH families were included in the analysis, which includes all the active GH families in the CAZy database (GH21, GH40, GH41, GH60, GH61, and GH69 have been deleted from the database). 15 carbohydrate esterase (CE) families were included in the analysis since the CE10 family was withdrawn. 26 polysaccharide lyase (PL) families were included in the analysis (PL19 has been deleted). All 13 auxiliary activities (AA) families were included. For families having subfamily classification (AA1, AA3, AA5, GH13, GH30, GH43, GH5, GT2, PL1, PL10, PL11, PL12, PL14, PL15, PL17, PL2, PL21, PL22, PL3, PL4, PL5, PL6, PL7, PL8, and PL9), the predicted CAZymes were assigned into subfamilies by using 207 newly added subfamily HMMs in dbCAN v6.

## Results

### Read cleaning and assembly

The RNA-Seq read sets pooled from the 12 to 15 samples sequenced for each of the four species contained from 298 to 562 million reads (Table 1). After cleaning the reads by trimming adapter sequences, removing low-quality sequences, and ribosomal RNAs, between 267 million (*N. frontalis*) and 513 million (*P. rhizinflata*) reads remained per transcriptome. The pooled reads of each species were assembled into contigs. Over 96% of the cleaned reads mapped to these contigs (Table 1). The contig sequences were polished by finding and correcting discrepancies with the consensus of the aligned reads. This polishing slightly increased the number of reads that mapped to the contigs, especially for those reads that mapped to multiple locations.

**Table 1:** Yield of RNA-Seq reads from four species of anaerobic rumen fungi after cleaning and assembly.

**Species**	**Reads**	**After trimming (%)**	**After rRNA removal (%)**	**Mapped to polished contigs (%)**	**Mapped to final transcriptome (%)**
*Anaeromyces mucronatus*	538,784,287	94.0	92.8	92.4	90.1
*Neocallimastix frontalis*	298,777,750	99.7	89.4	86.5	85.5
*Orpinomyces joyonii*	298,232,228	99.6	97.4	97.2	94.4
*Piromyces rhizinflata*	561,826,160	93.5	91.3	91.0	87.9

The distribution of the number of reads mapped per contig had a long tail of contigs with low coverage; more than 99.5% of the alignable reads mapped to less than half of the contigs. Approximately 10% of the reads mapped to more than one contig, resulting in the protein sequences predicted from the ORFs having considerable overlap. Consequently, we removed the contigs with extremely low read coverage and clustered the remaining contigs, firstly by nucleotide sequence similarity and secondly by similarity of the proteins encoded by their ORFs. Clustering slightly decreased the total fraction of reads that mapped to contigs (Table 1), increased the fraction that mapped uniquely and substantially decreased the fraction that mapped to multiple locations. Any non-coding transcripts that may have been present in the assembled contigs were removed by the ORF clustering step.

### Transcripts and ORFs

The number of predicted transcripts ranged from 6,633 (*O. joyonii*) to 12,751 (*N. frontalis*) (Table 2). *N. frontalis* and *P. rhizinflata* had about 60% more ORF-clusters than *A. mucronatus* and *O. joyonii*. Alternative clustering of the contigs with Corset, a method that relies on shared multi-mapping reads and similarities in expression profiles (Davidson and Oshlack, 2014), gave similar counts of predicted transcripts. The combined lengths of the transcripts were between 10.6 and 19.6 Mb per transcriptome (Table 2). Over 98% of the transcripts contained a single ORF with the remaining 2% being composed of two or three ORFs. The assembled transcriptomes of *A. mucronatus* and *O. joyonii* had substantially fewer transcripts than the transcriptomes of *N. frontalis* and *P. rhizinflatus* (Table 2). These two species also had fewer transcripts than were predicted for related species with assembled genomes, *A. robustus* (Haitjema et al., 2017) and *P. ruminatium* (Youssef et al., 2013). These discrepancies suggest that our transcriptome assembly may have missed some transcripts in *A. mucronatus* and *O. joyonii*. To investigate this possibility, we mapped the RNA-Seq reads of *A. mucronatus* and *O. joyonii* to the predicted transcripts of *A. robustus* and *P. ruminatium* C1A, respectively. Most of the *A. robustus* transcripts with an assembled homolog in *A. mucronatus* were covered by reads from A. mucronatus. In contrast, there were few *A. mucronatus* reads that mapped to the *A. robustus* transcripts that did not have an *A. mucronatus* homolog indicating that these transcripts had not been expressed in *A. mucronatus*.

**Table 2:** Assembled transcript statistics arising from RNA-seq reads generated from four species of anaerobic fungi.

**Species**	**Contig count**	**Total length**	**Median contig length**	L50^*^	**ORFs per contig^†^**			**Median ORF length**	**AT %**
					**1·····**	**2**	**3**		
*Anaeromyces mucronatus*	8,450	13,224,613	1,309	1,897	8,448	170	9	1,182	72.2
*Neocallimastix frontalis*	12,751	16,330,830	1,087	1,539	12,750	152	13	979	70.3
*Orpinomyces joyonii*	6,633	10,602,950	1,365	1933	6,564	66	3	1,227	71.6
*Piromyces rhizinflata*	12,160	19,564,255	1,310	2,005	11,955	197	7	1167	72.8

* *50% of the assembled bases were in contigs of this length or longer*.

† *Only ORFs of 210 bases or longer were counted*.

Transcripts in *A. robustus* that did have reads from *A. mucronatus* mapping to them despite the absence of these assembled transcripts in the *A. mucronatus* transcriptome showed a discontinuous pattern of coverage that was localized to short regions of 30–50 bases in the read. Outside of these localized regions there was no homology between the mapped reads and the transcript. These regions showed a high level of sequence identity localized in this region despite low identify over the rest of the transcript. A similar result was obtained whem comparing the *O. joyonii* and *P. ruminaintium* C1A. We interpret these as spurious mappings to short sequences that are common between *A. mucronatus* and *A. robustus*, and between *O. joyonii* and *P. ruminaintium* C1A, not as evidence of an unassembled *A. mucronatus* and *O. joyonii* transcripts.

### Transcriptome quality and completeness

Final transcriptomes were scored using TransRate (Smith-Unna et al., 2016). This tool combines measures of read mapping quality and completeness and uniformity of mapped read coverage to assign a score to each transcript and the overall transcriptome. The transcriptomes of *A. mucronatus, O. joyonii*, and *P. rhizinflata* had TransRate scores in the range 0.57–0.60 with very few low-scoring transcripts, indicating that these transcriptome assemblies were of high quality (Table 3). The transcriptome of *N. frontalis* had a lower assembly score and more low-scoring transcripts than the other three but had a higher score than the previously published *Piromyces* sp. “*finn*” transcriptome (Solomon et al., 2016). The lower score for the *Piromyces sp. “finn”* transcriptome is primarily due to the presence of transcripts with incomplete read coverage. The *P. sp. “finn”* transcriptome contained the most contigs with full-length ORFs, although 23% of these ORFs were low-scoring contigs. Both *Piromyces* species had more full-length ORFs in high-quality contigs than the other three species.

**Table 3:** Transcriptome quality and completeness measures.

**Species**	**Busco completeness (%)**	**TransRate Score**	**Concordantly mapped reads (%)**	**Low-scoring transcripts (%)**	**Full-length ORFS**	**High-scoring full length ORFs**
*Anaeromyces mucronatus*	73	0.60	94	1.7	5,037	4,971
*Neocallimastix frontalis*	56.4	0.50	78	9.9	6,472	6,022
*Orpinomyces joyonii*	61.6	0.57	94	0.7	4,270	4,258
*Piromyces rhizinflata*	76.3	0.58	93	4.4	7,428	7,187
*Piromyces sp. “finn”*	77	0.26	68	23	9,099	7,415

To estimate the fraction of genes represented in the transcriptomes, we assessed the presence of 1283 genes expected to be found as a single copy in every fungal genome with the Benchmarking Universal Single-Copy Orthologs (BUSCO) tool (Simão et al., 2015). Complete homologs of 60–77% of these genes were found in the assembled transcriptomes; 11–15% were not detected and 12–25% were incomplete (Table 3). In comparison, the previously reported transcriptome assemblies from *Piromyces* sp. E2 and *Pecoramyces* sp. C1A were missing 32% and 38% of the BUSCO genes (respectively). In contrast, the recent transcriptome from *Neocallimastix californiae* had excellent recovery of BUSCO genes with only 10% missing. Due to the limited number of conditions and substrates that the fungi were grown on, we do not expect all of the genes encoded in the genome to be expressed.

### Functional annotation of predicted proteins

Analysis of functional annotation for predicted proteins resulted in hits for 49–57% of the proteins in the four anaerobic fungi examined in this study (Table 4). The remaining proteins either had no hits in the database or matched hypothetical proteins of unknown function. Searching for functional sites and domains can provide additional insight into the function of unknown or hypothetical proteins (Mitchell et al., 2015). InterproScan identified functional sites and domains in 74–80% of the predicted proteins (Table 4). When combining results from both BLASTP search and InterProScan, 19–24% of the proteins from the four anaerobic fungal transcriptomes did not generate any hits.

**Table 4:** Number of proteins annotated by BLASTP search against NCBI's nr and SwissProt databases and InterPro domain prediction.

**Species**	**Total predicted proteins**	**Matched to proteins of known function**	**Matched to proteins of unknown function**	**No BLASTP hit**	**Have domains from InterProScan**
*Anaeromyces mucronatus*	8640	4734 (55%)	767 (9%)	3139 (36%)	6832 (79%)
*Neocallimastix frontalis*	12948	6415 (50%)	1078 (8%)	5435 (42%)	9570 (74%)
*Orpinomyces joyonii*	6709	3827 (57%)	590 (9%)	2288 (34%)	5361 (80%)
*Piromyces rhizinflata*	12379	6004 (49%)	1085 (9%)	5290 (43%)	9221 (74%)

**Figure 1:**
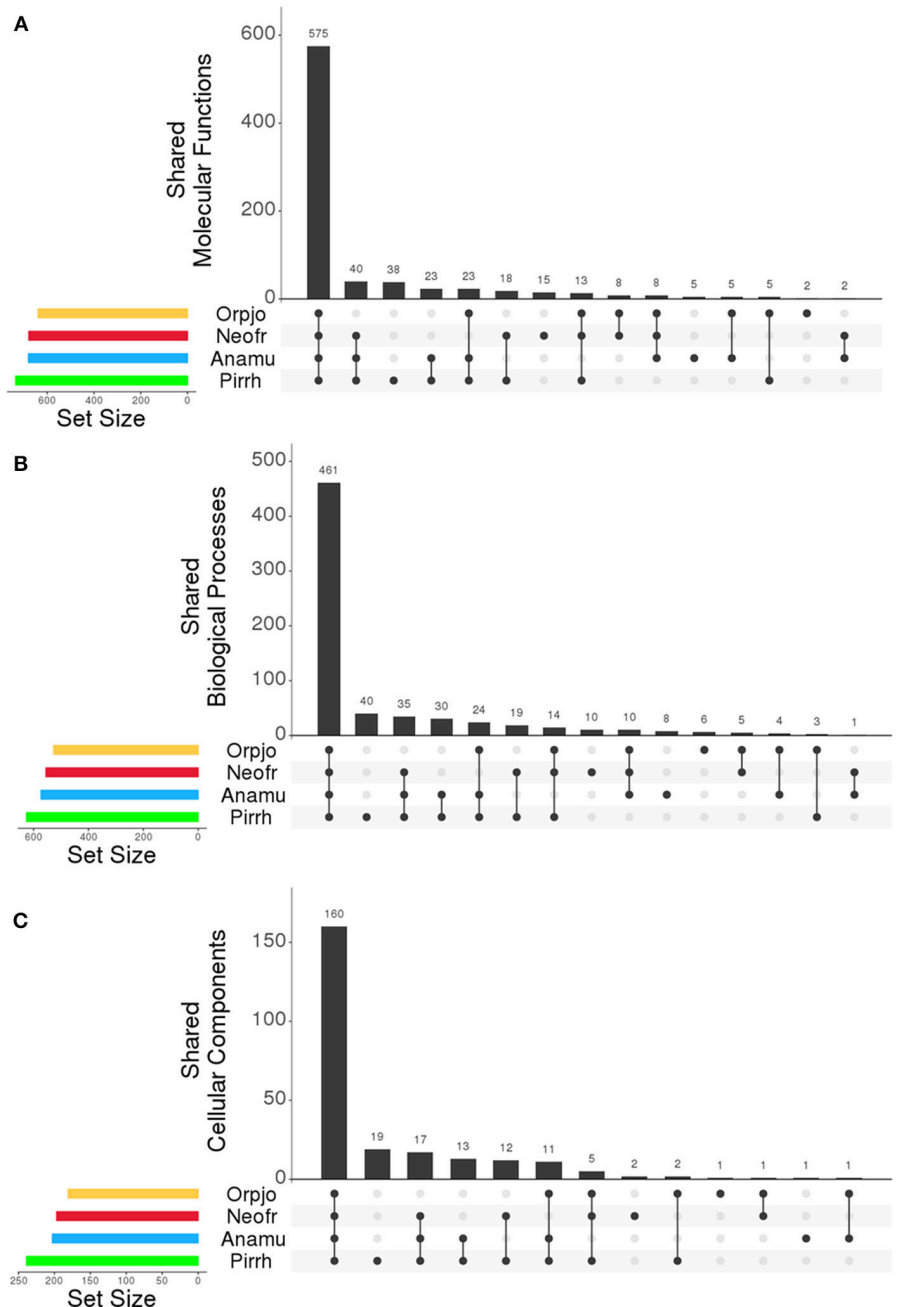
Comparison of the **(A)** molecular functions, **(B)** biological process, and **(C)** cellular components assigned to transcripts expressed by *O. joyonii* (Yellow)*, N. frontalis* (Red)*, A. mucronatus* (Blue), and *P. rhizinflata* (Green). Set size indicates the total number of GO functions identified in each organism within each category. The bars indicate the number of GO functions that are shared in the organisms being compared as indicated along the X-axis of the plot. Figure was generated with R using UpSet (Lex et al., 2014).

**Figure 2:**
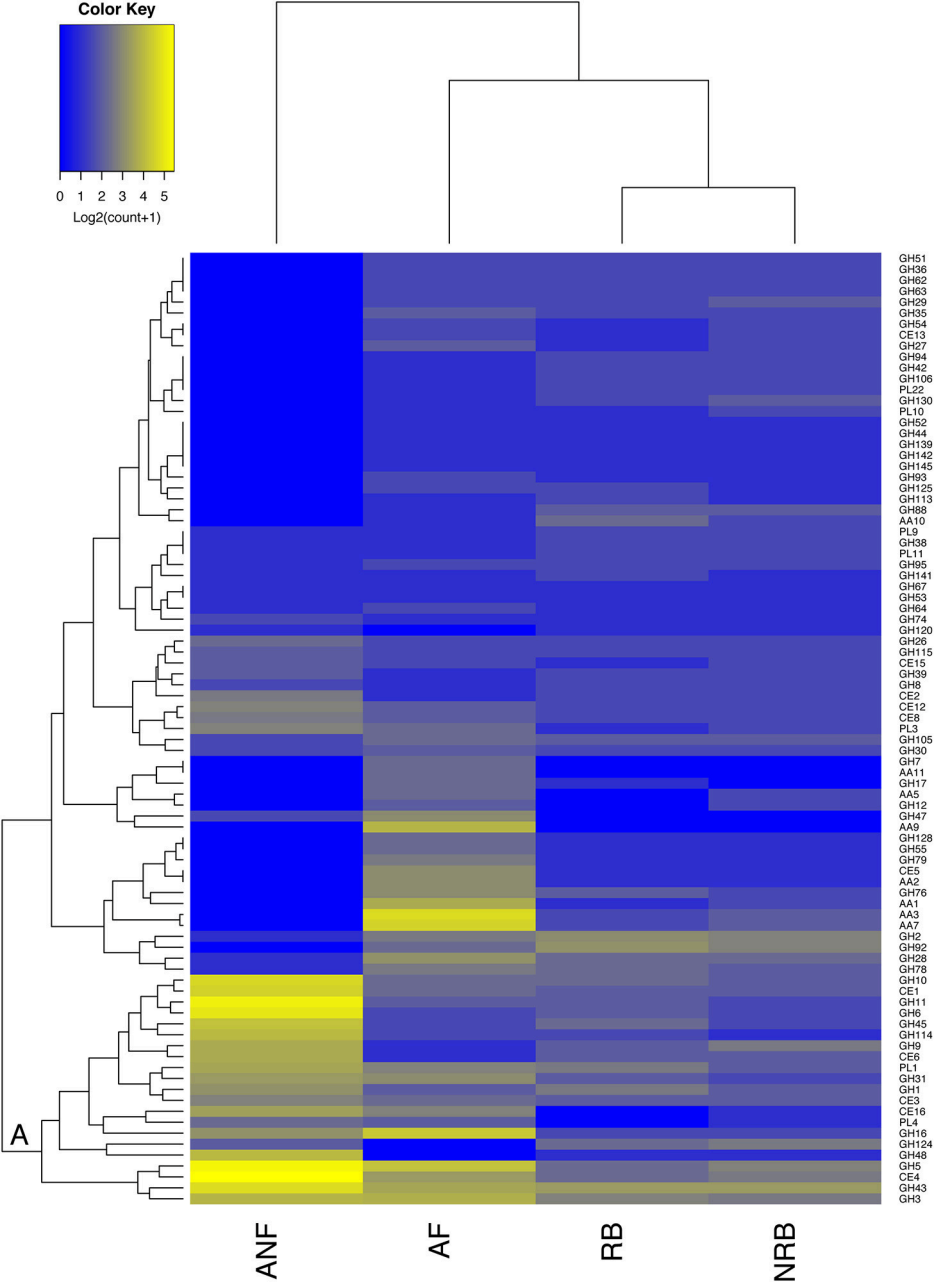
Comparison of the abundance of 87 CAZyme families expressed by anaerobic fungi (ANF) with their abundance in aerobic fungi (AF), non-rumen bacteria (NRB), and rumen bacteria (RB). The mean abundance of each CAZy family was calculated according to description in the materials and methods and was log2 transformed before generating a heatmap in R. CAZy families with high and low abundance are shown in blue and yellow, respectively. Hierarchical clustering on the vertical axis is based on the mean abundance of each CAZy family included in the analysis. Hierarchical clustering of the horizontal axis is based on the similarity among the distribution of these 87 CAZy modules among organism groups.

The program Interproscan was used to identity putative protein function using gene ontology (GO) classification scheme and the detection of pfam domains in the transcripts. The GO framework links gene products to biological function. A summary of the GO terms for each fungi can be found as Supplementary Datasheets 5–8. The most prevalent GO functions were consistent across species, showing small differences in the proportion of proteins that were found in each class. In all species, the three most prevalent molecular functions were: protein binding (GO:0005515), ATP binding (GO:0005524), and catalytic activity (GO:0003824). Cellulose binding (GO:0030248) was also an abundant function found in all species (Figure 2). The most abundant biological processes in the fungi were: carbohydrate metabolism (GO:0005975), protein phosphorylation (GO:0006468), transport (GO:0006810), oxidation-reduction (GO:0055114), proteolysis (GO:0006508), and translation (GO:0006412). All four species in this study had a significant proportion of their proteins associated with cellulose catabolism (GO:0030245). Proteins showed a similar distribution of cellular locations in all four fungi. The number of GO molecular functions, biological processes, and cellular components that were shared between all of the organisms can be seen in Figures 1A–C, respectively. All four genera of fungi shared the vast majority of the GO functions that were identified indicating a high level of similarity between them. We also examined the distribution of pfam domains that were identified in the fungal proteins. In all fungi the five most abundant domains were: Anykrin repeats (PF12796), cellulose or protein binding domain (PF02013), protein kinase domain (PF00069), CotH domain (PF08757), and chitin binding domain (PF00182). Among proteins involved in carbohydrate metabolism, the most abundant PFAM domains were: cellulose or protein binding domain (PF02013), chitin binding domain (PF00182) and fungal cellulose binding domains (PF00734). PFAM domains associated with the glycosyl hydrolase families: GH1, GH3, GH5, GH6, GH8, GH10, GH11, GH43, GH45, and GH48 were also abundant. Like the distribution of GO functions, there was a high similarity in the types of pfam domains identified in the transcripts in all four genera of anaerobic fungi.

### Comparative analysis of genes encoding carbohydrate active enzymes expressed by anaerobic fungi

The presence of an extensive array of genes encoding enzymes involved in carbohydrate metabolism in the anaerobic fungi is now well-documented (Youssef et al., 2013; Couger et al., 2015; Solomon et al., 2016). To further elucidate the mechanisms of lignocellulose digestion by these four anaerobic fungi, a detailed analysis and comparison of the complement of Carbohydrate active enzyme genes (CAZome) expressed on several carbon sources was undertaken. All four species of anaerobic fungi expressed a large number of genes encoding proteins containing CAZyme modules (GH, CE, GT, PL), as well as a number of domains of unknown function appended to putative carbohydrate binding modules (CBMs) (Table S3). The percentage of all transcripts that were identified as predicted CAZymes ranged from 8.1 to 11.2% of the transcriptome in each species (Table 5). The percentage of transcriptome encoding proteins with CAZy domains was roughly equal in *A. mucronatus* (8.1%), *N. frontalis* (9.0%), and *P. rhizinflata* (8.9%) but was significantly higher in *O. joyonii* (11.2%). Of the predicted CAZymes identified in the fungal transcripts, 56–60% had an identifiable catalytic CAZy domain (Table 5). In the four anaerobic fungi, 40–44% of the contigs encoding CAZymes had one or more known carbohydrate binding modules (CBMs), but no identifiable catalytic CAZy domain (Table 5). The most commonly observed CBMs were CBM10, CBM18, and CBM1 (Table S3).

**Table 5:** Comparison of CAZome classes of four species of anaerobic fungi.

**Species**	**CAZymes**	**Percentage of Transcriptome (%)**	Glycosyl-transferase^*^	**No CBM**	**CBM with known CAZy domain**	**CBM without known CAZy domain**
*Anaeromyces mucronatus*	700	8.1	77 (11.0%)	140 (20.0%)	174 (24.9%)	309(44.1%)
*Neocallimastix frontalis*	1167	9.0	119 (10.2%)	276 (23.7%)	262 (22.5%)	510 (43.7%)
*Orpinomyces joyonii*	749	11.2	68 (9.1%)	164 (21.9%)	215 (28.7%)	302 (40.3%)
*Piromyces rhizinflata*	1100	8.9	126 (11.5%)	268 (24.9%)	225 (20.5%)	481 (43.7%)

* *The percentages are the relative percentage of the whole CAZome that each category represents*.

Across all four fungi, 65–73% of CAZymes belonged to families with members known to cleave bonds within plant cell wall polysaccharides (Table 6). Differences in CAZome profiles suggests that there may be some degree of specialization among the species (Table 6, Figure 2). *N. frontalis* had the largest overall CAZome, however, it appears to contain the least degree of specialization as compared to the other fungi in this study. In general, most of the CAZymes targeting the plant cell wall were putative cellulases with *O. joyonii* containing the highest number of cellulases of the fungi examined (Table 6). Additionally, each species had a large number of hypothetical proteins that may play a role in hemicellulose modification. Xylanases, most highly represented by members of GH10 and GH11, were present at similar levels (13.9–17.4%) in all four fungi. *A. mucronatus* appears to have dedicated the largest percentage of its CAZome to xylan saccharification. Compared to enzymes targeting cellulose, xylan and hemicellulose, enzymes targeting pectin carbohydrates were the least numerous in all four fungi (Table 6). *P. rhizinflata* had almost twice as many putative pectinases compared to the other three anaerobic fungi.

**Table 6:** The number of transcripts encoding CAZymes in anaerobic fungi that target plant cell wall carbohydrates^*^.

**Species**	**Xylan^a^**	**Cellulose^b^**	**Pectin^c^**	**Hemicellulose sidechains and modifications^d^**
*Anaromyces mucronatus*	40 (13.9)	66 (23.0)	20 (7.0)	61 (21.0)
*Neocallimastix frontalis*	77 (15.9)	135 (28.0)	23 (4.8)	83 (17.0)
*Orpinomyces joyonii*	50 (14.6)	112(32.7)	23 (6.7)	67 (19.3)
*Piromyces rhizinflata*	75 (17.4)	113 (26.2)	41 (9.5)	65 (14.8)

* Values in brackets represent the percentage of GHs, CEs, and PLs transcripts that have a predicted function involved in the degradation of xylan, cellulose, pectin or hemicellulose debranching. ORFs that had unknown function or no hit were not included in the table.

a GH8, GH10, GH11, GH141;

b GH1, GH5_1, GH5_4, GH5_5, GH6, GH9, GH45, GH48, GH74;

c CE8, CE12, GH105, PL;

d *CE1, CE2, CE3, CE4, CE6, CE15, GH43, GH53, GH67, GH95, GH115, GH120*.

The most diverse group of CAZymes in the anaerobic fungi were the GHs (Table S3). Approximately half of these were also associated with one or more CBM. In order of highest to lowest abundance, the 10 most abundant families in all fungi are: GH11, GH43, GH5, GH10, GH114, GH45, GH6, GH3, GH9, and GH13. With the exception of GH13 and GH114 all of these families are involved in plant cell wall deconstruction. This finding is consistent with the known ability of anaerobic fungi to digest lignocellulose with high efficiency (Youssef et al., 2013; Couger et al., 2015; Solomon et al., 2016). The three most abundant CE families in all anaerobic fungi were CE1, CE4, and CE6. Additional carbohydrate esterase families found included CE8, CE12, and CE16. All four fungal species also possessed members of three families of putative polysaccharide lyases (PL1, PL3, and PL4) that may have activity specific for pectin (Table S3). In contrast to the glycosyl hydrolases, the number of glycosyl transferases was comparatively small, ranging from 7 to 10% of the CAZome. The most abundant GT families were GT2, GT34, GT71, GT4, GT17, and GT39. These GTs are involved in various metabolic processes including cell wall biosynthesis, chitin synthesis, and glycosylation (Lombard et al., 2014). Expansin-like proteins were also identified in low abundance with two contigs in *A. mucronatus* and five in *N. frontalis*.

### Comparative analysis of CAZy families present in anaerobic fungi (ANF), aerobic fungi (AF), rumen bacteria (RB) and non-rumen bacteria (NRB)

We compared the CAZome composition of the transcriptomes of six anaerobic fungi (ANF) with the genomes of 104 aerobic fungi (AF), 49 rumen bacteria (RB), and 53 non-rumen bacteria (NRB). This subset of microbes were selected based on their ability to digest complex carbohydrates within plant cell walls including: cellulose, hemicellulose, pectin, and xylan and oligomers that result from the breakdown of these polymers For each organism the number of encoded proteins in each CAZy family was determined (Table S4). Glycosyl transferases were not included in the analysis because they are not involved in the degradation of carbohydrate substrates.

Amongst 128 GH, 15 CE, and 23 PL families examined in this analysis, 55 were found in the anaerobic fungi. To compare the strategies to degrade lignocellulose that have evolved in the distinct environments inhabited by these groups of microbes, we examined the mean number of GH, CE, PL, and AA families encoded in the four organism groups (Figure 2). We considered those families with known activities that target carbohydrates within the plant cell wall. Hierarchical clustering of these CAZy families based on their mean abundance in each organism group found 14 families that were present in much higher amounts in the anaerobic fungi compared to the other microorganisms examined. These 14 families clustered intoone large clade consisting of enzyme families that primarily specialize in deconstruction of the plant cell wall (Figure 2). The families that are found in higher abundance in the anaerobic fungi within this clade are: GH11, GH6. GH10, GH45, GH114, GH9, CE6, GH1, GH48, CE1, GH43. GH5, and CE4. None of these enzyme activities were completely exclusive to the anaerobic fungi.

There were significant differences between groups in the enzymes involved in plant cell wall carbohydrate digestion (Figure 2). There were more differences between the CAZome of anaerobic fungi and bacteria. There were 126 families that were identified in the rumen bacteria included in the analysis, and 144 families in the non-rumen bacteria that were not identified in the anaerobic fungi. Comparing the CAZome composition of aerobic and anaerobic fungi revealed 99 CAZy families in aerobic fungi were not found in the anaerobic fungi. One of the most glaring differences between anaerobic and aerobic fungi is the extensive use of oxidative enzymes to digest lignocellulose by aerobic fungi. Additionally, the GH7 family of β-1,4-endoglucanases and non-reducing end cellobiohydrolases were exclusive to aerobic fungi. The absence of these enzymes in anaerobic organisms has been reported previously (Youssef et al., 2013; Couger et al., 2015; Solomon et al., 2016; Haitjema et al., 2017). Our analysis supports previous findings that the anaerobic fungi have evolved a highly specialized CAZome for degrading lignocellulose and that the majority of the CAZy families that are expressed are concentrated in a small number of enzyme families (Figure 2). In contrast, the aerobic fungi encode a smaller number of CAZy enzymes that are distributed over a wider range of CAZy families.

The anaerobic fungi possess an unusually high number of transcripts that encode CBMs which are thought to participate in cellulosome-mediated carbohydrate digestion (Youssef et al., 2013; Couger et al., 2015; Solomon et al., 2016; Haitjema et al., 2017). Our comparative analysis of CBM families supports the findings of these previous studies. Although all of the groups included in our analysis encode proteins associated with CBMs that target a wide range of carbohydrates including cellulose, xylan, chitin, and starch, the absolute number of CBMs that were identified in anaerobic fungi exceeded all other organism groups by at least 5-fold (Table S4). In addition, whereas CBMs were found ubiquitously in the anaerobic fungi they were much less frequently observed in microorganisms within the other groups.

### CAZy families with extended gene numbers in anaerobic fungi

**Figure 3:**
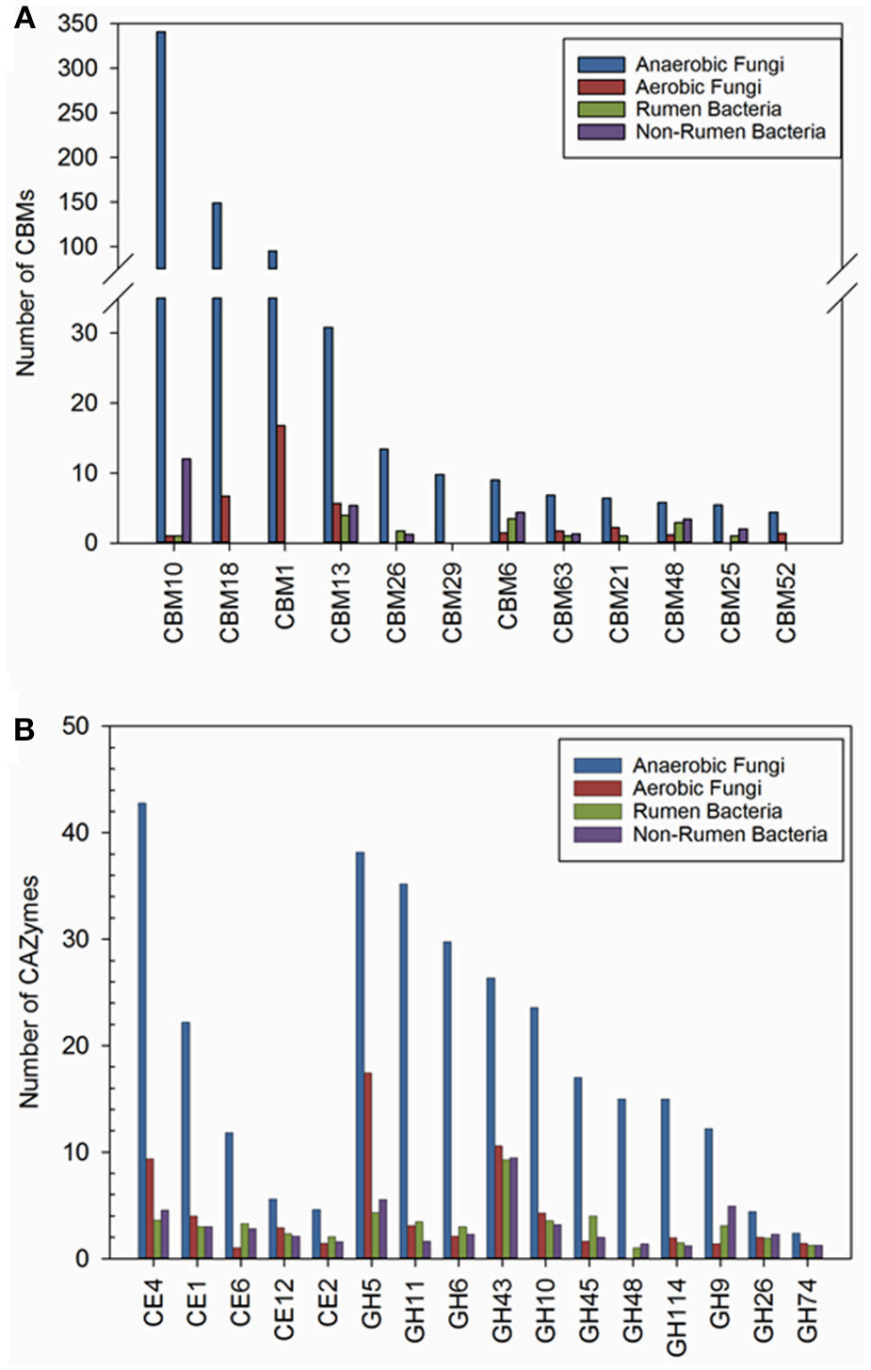
**(A)** Carbohydrate binding modules and **(B)** carbohydrate active enzyme families that were found to be expanded the anaerobic fungi (ANF) compared to aerobic fungi (AF), rumen bacteria (RB), and non-rumen bacteria (NRB). Counts represent the mean abundance of the CAZy family and was calculated according to description in the materials and methods. CAZy families with a mean abundance that was at least 2-fold higher in the anaerobic fungi compared to all other groups of microorganisms were considered to be expanded.

We also sought to identify CAZy families that have been expanded in anaerobic fungi (ANF) compared to aerobic fungi (AF), non-rumen bacteria (NRB) and rumen bacteria (RB). Our comparative analyses identified 14 CAZy families and 11 CBMs that were at least 2-fold higher in abundance in ANF compared to any other organisms (Figure 3A). There has been a clear expansion in the use of CBMs for cellulose, chitin and starch digestion in anaerobic fungi (Figure 3A). Furthermore, 14 enzyme families involved in lignocellulose digestion were expanded in anaerobic fungi (Figure 3B). Previously, these families have been found to be highly abundant both in the rumen (Dai et al., 2015) and in pure cultures of anaerobic fungi grown on lignocellulose (Couger et al., 2015; Solomon et al., 2016). The four families with cellulose activity, GH6, GH9, GH45, and GH48, seemed to be present in low copy number or low abundance in most of the analyzed aerobic fungi and rumen bacteria. GH6 and GH48 proteins have non-reducing and reducing end cellobiohydrolase (EC 3.2.1.176) activity, respectively. Both of these enzymes are associated with the anaerobic fungal cellulosome and their encoding genes are highly expressed within the rumen environment (Dai et al., 2015; Haitjema et al., 2017). All of the expanded families that we have identified are conserved in all of the species of anaerobic fungi examined using transcriptomics (Table S5). Esterase families CE1, CE4, and GH43 were found in the majority of organisms examined. In contrast, expansion of PL families in anaerobic fungi was not observed. The presence of pectate lyases was universal amongst the anaerobic fungi whereas, these enzymes were much less commonly found in the other groups of organisms, particularly bacteria (Table S5).

## Discussion

We have reported four new high quality transcriptomes from diverse species within the phylum Neocallimastigomycota. These data contribute to the growing database of anaerobic fungal sequences and provide information that can be used by the scientific community to expand our understanding of the biology of these unique organisms. The conclusions that can be drawn from a transcriptomic study rests on the quality of the assembled transcripts. As more and more transcriptomes from non-model microorganisms have been assembled, awareness of potential pitfalls of de novo transcriptome assembly has grown (MacManes, 2016; Cabau et al., 2017). Different assemblers have different biases and fail to assemble different transcripts. Artifacts such as chimeras and partial transcripts are produced. Assemblies are especially sensitive to the length of the kmers used to construct their de Bruijn graphs; short kmers allow the assembly of low-abundance transcripts that are missed with longer kmers, and longer kmers support the more accurate assembly of high-abundance transcripts. Assembly pipelines that combine the output of different assemblers and assemblies using different kmer values have been shown to produce more accurate transcriptomes than their component assemblers operating individually (MacManes, 2016; Cabau et al., 2017). To minimize the introduction of these artifacts, we have chosen an assembler that uses a range of kmer lengths within each run. In addition we filtered and clustered the raw assemblies to produce non-redundant sets of transcripts and have critically evaluated their quality. Lastly, we have integrated our results with the results of other transcriptomic and genomic studies of the Neocallimastigomycota (Wang et al., 2011; Youssef et al., 2013; Couger et al., 2015; Solomon et al., 2016; Haitjema et al., 2017; Mondo et al., 2017).

Even when a high quality transcriptome or genome is generated, it may be difficult to provide a functional annotation of organisms from environmental sources if there is a lack of characterized homologs available within databases. In these cases, it can be helpful to search for the presence of functional domains within the transcripts. The presence of these domains can allow one to infer the biological function of unknown or hypothetical proteins (Mitchell et al., 2015). Using this approach, we were able to predict the function of 74–80% of the fungal proteins by detecting pfam domains and GO terms. The GO classification scheme provides a means to describe the function of a particular gene and classifies it based on the predicted molecular activity of the gene product (molecular function), where the gene product is active (cellular component) and the pathways/processes the gene product activity is associated with (Ashburner et al., 2000).

The number of assembled transcripts varied from a low of 6633 in *O. joyonii* to a high of 12751 in *N. frontalis*. Despite the relatively low number of expressed transcripts in *O. joyonii* and *A. mucronatus* compared to *P. rhizinflata* and *N. frontalis*, these smaller transcriptomes appear to be complete representations of the genes expressed by these fungi under the growth conditions we sampled. This conclusion is supported by the lack of any evidence for additional transcripts from cross-mapping the RNA-Seq reads of *O. joyonii* and *A. mucronatus* to the predicted transcripts of *P. ruminatium* and *A. robustus*, respectively. In addition, very high fractions of the RNA-Seq reads of these two species aligned to their assembled transcripts (Table 3). The implication that these fungi require only 8450 or fewer transcribed genes for growth, even on complex lignocellulosic materials like barley and rice straws, is further supported by the relatively high fractions of Busco gene transcripts found in their transcriptomes (Table 3). There may be other genes in the genomes of *O. joyonii* and *A. mucronatus* that were not expressed in our culture conditions and it would be interesting to test a wider variety of conditions to see if transcription of more genes could be detected. It is interesting to note that both *O. joyonii* and *A. mucronatus* are polycentric fungi whereas *N. frontalis* and *P. rhizinflata* are monocentric however, it is currently unclear why the latter two fungi express more genes under similar growth conditions and what advantage this would provide to these organisms.

Despite differences in the number of genes expressed between fungi, a comparative analysis of the function of the transcripts encoding proteins expressed by all four anaerobic fungi shows a high degree of similarity amongst their functionality (Figure 1). The similarity in the function of these transcripts suggests that a core set of genes t has been maintained throughout the evolution of these microorganisms. Comparative analysis of the genomes of *A. robustus, N. californiae, Pecoramyces ruminantium, Piromyces finis*, and *Piromyces* sp. E2 also showed high functional conservation between these organisms (Youssef et al., 2013; Edwards et al., 2017). These previous studies, together with our functional analysis, allows us to hypothesize that the fungi inhabiting the rumen have maintained a core set of genes that encode functions that are specialized for colonizing lignocellulosic substrates in the rumen.

### A large, functionally redundant cazome is conserved across both genus and species in the phylum neocallimastigomycota

The recent increase in interest surrounding the Neocallimastigomycota is related to their potential value in the industrial saccharification of lignocellulose. Several studies have shown that anaerobic fungi have developed multiple approaches to degrade recalcitrant lignocellulosic material and use a combination of invasive rhizoidal growth, expression of expansin proteins, and the production of a range of powerful polysaccharide degrading enzymes (Youssef et al., 2013; Couger et al., 2015; Solomon et al., 2016; Haitjema et al., 2017; Henske et al., 2017). Indeed, our results show that all four genera of anaerobic fungi express a diverse array of CAZyme genes encoding enzymes having functionally redundant activities (Couger et al., 2015; Solomon et al., 2016; Henske et al., 2017). The transcriptomes of all four fungi examined in this study consisted of 8–11% CAZyme transcripts. Many of these putative cell wall degrading enzymes have low levels of sequence identity to proteins that have been characterized to date and thus represent a potentially rich source of novel CAZymes and undescribed catalytic activities (Couger et al., 2015; Solomon et al., 2016; Jones et al., 2017). Our comparative analysis of CAZome composition in diverse lignocellulose degrading microorganisms found significant expansion of CBMs and CAZy families that are known to be involved in plant cell wall digestion in the anaerobic fungi. Of note, many of the CAZy families that are expanded in anaerobic fungi were not widely found in the genomes of the rumen bacteria we examined. The expansion of these families in the rumen fungi may unlock new substrates and reduce direct competition for resources with rumen bacteria, which are present in higher numbers and account for a higher proportion of the biomass within the rumen.

Comparative CAZome analysis reveals that there may be some degree of metabolic specialization between the fungi. Differences in the relative proportions of CAZyme transcripts predicted to be involved in cellulose, hemicellulose, and pectin saccharification could help decrease direct competition for finite resources in the rumen and facilitate the co-existence of multiple genera of anaerobic fungi in the gut. A study by Bootten et al. (2011) found that different genera of anaerobic fungi were able to colonize and degrade lignified secondary plant cell walls with differing efficiency and specificity. *N. frontalis* and *P. communis* were highly efficient at degrading all plant cell wall structures whereas *Caecomyces communis* preferentially degraded xylem tissues and was much less efficient than the other two genera studied (Bootten et al., 2011). The results of this study provide some support that there is at least some degree of specialization among species of anaerobic fungi.

Comparing our results to that of previous studies, we find that the most prominent CAZy families are similar (Couger et al., 2015; Solomon et al., 2016; Haitjema et al., 2017). However, there are differences in the number of transcripts expressed by these species, and the relative abundance of the CAZy families. These differences most likely arise due to variation in the CAZome between closely related species and/or strains within the phylum Neocallimastigomycota, variation in the methods used for transcriptome assembly and bioinformatics analysis, and the nature of the fungal growth conditions and carbon sources used.

Several GH families including GH6, GH9, GH45, and GH48 were found in high abundance in all species and have synergistic activities involved in cellulose digestion. The most abundant enzymes involved in hemicellulose digestion were GH10, GH11, and GH43. All of these families have undergone gene expansion in the anaerobic fungi. Additionally, the expansion of esterases involved in deesterfication of hemicellulose may increase the accessibility of the xylan backbone for depolymerisation or disrupt covalent linkages between hemicellulose and the lignin network. Unlike aerobic fungi, oxidative catalysts that degrade lignin are not present in the strict anaerobic environment of the rumen. The expansion of esterases within the anaerobic fungi may help overcome their lack of oxidative enzymes and improve the efficiency of lignified plant cell wall digestion in the rumen.

The ability of anaerobic fungi to effectively degrade pectin may be important for their role in the primary colonization and digestion of plant material in the rumen (Edwards et al., 2008). Anaerobic fungi have been shown to colonize plant material in the rumen within minutes, where the hyphae physically disrupt the plant cell wall and increase the surface area for digestion by potent carbohydrate degrading enzymes (Orpin and Joblin, 1997). The distribution of PLs within anaerobic fungi is interesting in two ways. Firstly, unlike aerobic fungi and bacteria, the PL families that are encoded by the rumen fungi exclusively target carbohydrates associated with pectin. Secondly, whereas the presence of PLs is not a widely observed in aerobic fungi and bacteria, the PL families PL1, 3, 4 are universally conserved between the species of anaerobic fungi examined to date. GH28 polygalacturonadases are also important in pectin digestion but are not found in anaerobic fungi. GH28 enzymes hydrolyze α-1,4 linked polygalacturonic acid (Abbott and Boraston, 2007). All GH28 enzymes have activity within a narrow pH range of pH 3–5, whereas PLs tend to have pH optima that range from close to neutral to more alkaline pH (Murphy et al., 2011). Fungi that inhabit acidic environments have more GH28 enzymes than PLs, while fungi that thrive in neutral pH have more PLs than GH28 enzymes (Berka et al., 2011). The lack of GH28s and universal conservation of PLs in rumen fungi is consistent with the physiochemical environment present in the rumen and implies that the cleavage of polygalacturonic acid occurs exclusively through a lytic mechanism or there are unknown enzymes that possess polygalaturonidase activity in the rumen.

One of the most abundant CAZy families seen in all of the anaerobic fungi was the GH114 family. In one case, endo-α-1,4-polygalactosaminidase (EC 3.2.1.109) has been demonstrated in a GH114 protein however, there is little that is known about the biological function of this family (Tamura et al., 1995). In aerobic fungi, α-1,4 linked galactosaminogalactan is a secreted cell wall exopolysaccharide and is important for virulence, cell wall structure, adhesion, and biofilm formation (Lee et al., 2015; Hervé et al., 2016). If α-1,4 linked galactosaminogalactan functions similarly in anaerobic fungi these enzymes could be important for regulating these possesses. Alternatively, the GH114 in anaerobic fungi may possess a previously unknown catalytic function. Biochemical characterization of this abundant enzyme family is needed to address the role that these enzymes play in anaerobic fungi.

### Cbms play key role in carbohydrate breakdown throughout the phylum neocallimastigomycota

The anaerobic fungi are known to possess an unusually high number of genes encoding proteins containing CBMs (Haitjema et al., 2017). All four species of fungi examined in this study contained a large number of CBMs with 70–73% of the transcripts in the anaerobic fungal CAZome being associated with CBMs. The almost ubiquitous presence of CBMs indicates that these domains are central to the ability of these microbes to effectively utilize and compete for the carbohydrates found in their natural environment. The majority (44–49%) of the CBM modules associated with transcripts were CBM10 fungal dockerin domains. CBM1 and CBM18 domains were also abundant in all four fungi. CBM10 domains are hypothesized to be involved in the dockerin interaction in the anaerobic fungal cellulosome (Fanutti et al., 1995), CBM1s bind cellulose and are important in plant cell wall digestion (Lehtiö et al., 2003), and CBM18s target chitin (Schwelm et al., 2015). Similar results were obtained in previous transcriptomic and genomic studies in other species of anaerobic fungi (Couger et al., 2015; Solomon et al., 2016; Haitjema et al., 2017). The role of CBMs in potentiating CAZyme activity, and in linking diverse CAZymes within the fungal cellulosome, are likely critical to the survival of anaerobic fungi within the highly competitive rumen ecosystem. The fungal biomass in the rumen is relatively low compared to bacteria (Dehority and Orpin, 1997) and the use of CBMs to potentiate CAZyme activity (Abbott and van Bueren, 2014) may enable the anaerobic fungi to effectively compete with rumen bacteria for attachment to substrates. A high percentage of CAZyme transcripts identified in this study had a CBM domain but no known catalytic domain. The presence of CBM domains in these transcripts implies that their activities are related to carbohydrate metabolism and suggests they may possess novel catalytic domains in the regions flanking the CBM domain (Jones et al., 2017).

## Conclusion

This study provides additional insight into how anaerobic fungi have evolved to become specialists at breaking down the plant cell wall in the complex, strictly anaerobic rumen environment. Fungi in the rumen have maintained a core set of genes that contribute to the ability of these microbes to compete for resources within this environment. The expression of a large, diverse contingent of CAZy genes is a universal feature of the phylum Neocallimastigomycota. Furthermore, the ubiquitous association of CAZymes with CBMs undoubtedly aids these microbes in the competition for resources in the rumen. We hypothesize that the variability in the CAZome composition between different genera of anaerobic fungi alleviates direct competition for resources in the rumen and helps facilitate their co-existence. This variability may also be central to the ability of these microbes to effectively target plant cell walls with a wide range of chemical and structural heterogeneity. Our results add to the body of knowledge surrounding the mechanisms that the anaerobic fungi utilize to degrade lignocellulose and effectively compete for resources in the highly competitive rumen environment.

## Author contributions

RG cultured fungi, extracted RNA, analyzed data, and wrote manuscript. TN and IR contributed equally performing bioinformatic analysis and contributed to writing of the paper. JY and PW cultured fungi. DA contributed to data analysis. AT contributed to data analysis and writing of paper. TM contributed to data analysis and writing of paper.

### Conflict of interest statement

The authors declare that the research was conducted in the absence of any commercial or financial relationships that could be construed as a potential conflict of interest.
