# Supplementary material for: Application of Transcriptomics to Compare the Carbohydrate Active Enzymes That Are Expressed by Diverse Genera of Anaerobic Fungi to Degrade Plant Cell Wall Carbohydrates
Source: Front Microbiol. 2018 Jul 16;9:1581. doi: 10.3389/fmicb.2018.01581 (PMC6054980; doi:10.3389/fmicb.2018.01581)
Supplement: Supplementary file 9 [file Data_Sheet_9.docx]

Supplementary material legend:

Supplementary table S1: Composition of Lowes semi-defined media and the solutions required to prepare this media. The final volume of all solutions is 1 L.

Supplementary table S2: microbes included in comparative analysis of CAZy gene abundance in Anaerobic fungi, aerobic fungi, rumen bacteria and non-rumen bacteria

Supplementary table S3: number of each CAZy family in the transcriptomes of *Anaeromyces mucronatus, Neocallimastix frontalis, Orpinomyces joyonii,* and *Piromyces rhizinflata*

Supplementary table S4: Average counts and percentage abundance of CAZy families from our comparative analysis of CAZy families present in anaerobic fungi (ANF), aerobic fungi (AF), rumen bacteria (RB) and non-rumen bacteria (NRB)

Supplementary Data 1: Transcriptome annotations for genes in *Piromyces rhizonflata* transcriptome

Supplementary Data 2: Transcriptome annotations for genes in *Orpinomyces joyonii* transcriptome

Supplementary Data 3: Transcriptome annotations for genes in *Neocallimastix frontalis* transcriptome

Supplementary Data 4: Transcriptome annotations for genes in *Anaeromyces mucronatus* transcriptome

Supplementary Data 5: Distribution of Pfam domains identified in the transcriptomes of *Anaeromyces mucronatus, Neocallimastix frontalis, Orpinomyces joyonii,* and *Piromyces rhizinflata*

Supplementary Data 6: Distribution of Gene Ontology Molecular functions identified in the transcriptomes of *Anaeromyces mucronatus, Neocallimastix frontalis, Orpinomyces joyonii,* and *Piromyces rhizinflata*

Supplementary Data 7: Distribution of Gene Ontology Biological processes identified in the transcriptomes of *Anaeromyces mucronatus, Neocallimastix frontalis, Orpinomyces joyonii,* and *Piromyces rhizinflata*

Supplementary Data 8: Distribution of Gene Ontology Cellular components identified in the transcriptomes of *Anaeromyces mucronatus, Neocallimastix frontalis, Orpinomyces joyonii,* and *Piromyces rhizinflata*

Supplementary Data 9: Supplementary material legend
