# Supplementary material for: Application of Transcriptomics to Compare the Carbohydrate Active Enzymes That Are Expressed by Diverse Genera of Anaerobic Fungi to Degrade Plant Cell Wall Carbohydrates
Source: Front Microbiol. 2018 Jul 16;9:1581. doi: 10.3389/fmicb.2018.01581 (PMC6054980; doi:10.3389/fmicb.2018.01581)
Supplement: Supplementary file 10 [file Table_1.DOCX]

**Supplementary table S1**: Composition of Lowes semi-defined media and the solutions required to prepare this media. The final volume of all solutions is 1 L.

| Lowes semi-defined media (per 1 L) | PO_4_ solution | Macronutrient solution | Trace minerals solution^*4^ | Volatile fatty acid solution^*5^ | Vitamin mix |
| --- | --- | --- | --- | --- | --- |
| 75 mL PO_4_ solution | 4.5 g KH_2_PO_4_ | 9 g KCl | 0.25 g MnCl_2_-4H_2_0 | 6.85 mL Acetic acid | 0.25 g 1,4-naphthoquinone |
| 55 mL macronutrient solution |  | 9 g NaCl | 0.25 g NiCl_2_-6H_2_0 | 3 mL Propionic acid | 0.2 g Calcium-D-panthothenate |
| 10 mL Trace minerals solution |  | 7.5 g MgSO_4_-7H_2_0 | 0.25 g NaMoO_4_-2H_2_0 | 1.85 mL butyric acid | 0.2 g nicotinamide |
| 10 mL Volitile fatty acid solution |  | 3 g CaCl_2_ | 0.25 g H_3_BO_3_ | 0.55 mL 2-methylbutyric acid | 0.2 g riboflavin |
| 10 mL 0.1% w/v hemin^*1^ |  | 8.1 g NH_4_Cl | 0.2 g FeSO_4_-7H_2_0 | 0.47 mL isovaleric acid | 0.2 g thiamin |
| 1 mL 0.1% w/v resazurin^*2^ |  |  | 0.05 g CoCl_2_-6H_2_0 | 0.55 mL n-valeric acid | 0.2 g pyridoxine-HCl |
| 10 mL vitamin mix |  |  | 0.07 g NaSeO_3_ |  | 0.025 g biotin |
| 1 g tryptone peptone |  |  | 0.05 g NH_4_VO_3_ |  | 0.025 g folic acid |
| 0.5 g yeast extract |  |  | 0.025 g ZnCl_2_ |  | 0.025 g cyanocobalamin |
| 1.5 g PIPES buffer |  |  | 0.025 g CuCl_2_-2H_2_0 |  | 0.025 g para-aminobenzoic acid |
| 50 mL clarified rumen fluid^*3^ |  |  |  |  |  |
|  |  |  |  |  |  |

*1 Hemin is dissolved in 5 mM NaOH at a concentration of 0.1% w/v

*2 Resazurin is dissolved in water at a concentration of 0.1% w/v

*3 Rumen fluid can be left out of media however anaerobic fungi tend to grow significantly better when this is included.

*4Trace mineral solution must be made using 0.2 M HCl to dissolve components

*5 Volatile fatty acids are added to 700 mL of 0.2 M NaOH and the pH is adjusted to 7.5 with 1 M NaOH. The solution is then diluted with water to a final volume of 1 L.

*Preparation of anaerobic media*

- 1. Prepare enough liquid modified Lowes media supplemented with 1% w/v carbon source to make 3 x 100 mL media bottles per carbon source.
  2. Combine media components as indicated in Table 1 and adjust pH to 6.7.
  3. Add 50 ml 8% w/v Na_2_CO_3_ and adjust volume of media to 1 L with distilled H_2_0.
  4. Using the Hungate method of preparing anaerobic media, gently bubble reduced, anaerobic CO_2_ into the media and bring it to a boil with a bunson burner. Be careful not to boil the media over. When the media reaches a boil, add 0.1 g of cysteine to completely reduce it. The media should go from a pink colour to a clear or transparent light yellow due to the reduction of resazurin. The Hungate method utilizes a specialized apparatus that enables researchers to work with anaerobic cultures and media on the bench-top while maintaining strict anaerobic conditions. Readers are referred to the review by Wolfe for details of setting up and working with a Hungate system (Wolfe et al 2011)
  5. Using the Hungate apparatus, anaerobically dispense 100 mL of the reduced media into culture bottle containing the appropriate amount of carbon source to make the final concentration 1 g/100 mL
  6. Cap bottles containing reduced media and carbon source and autoclave.
  7. When media prepared as described above has cooled, inoculate the media with fungal mycelia and incubate at 39°C under anaerobic conditions for 72 hrs. Do not shake flasks as this increases the possibility of oxygen being introduced to the growth media.

**Reference:**

Wolfe R. 2011. Techniques for cultivating methanogens. Methods in Enzymology 494:1-22
